# Complete Genome Sequences of Two Astrovirus MLB1 Strains from Bhutanese Children with Diarrhea

**DOI:** 10.1128/genomeA.00485-13

**Published:** 2013-07-25

**Authors:** Takashi Matsumoto, Sonam Wangchuk, Kinlay Tshering, Takaaki Yahiro, Sangay Zangmo, Tshering Dorji, Karchung Tshering, Marcelo Takahiro Mitui, Akira Nishizono, Kamruddin Ahmed

**Affiliations:** Department of Microbiology, Faculty of Medicine, Oita University, Yufu, Japana; Public Health Laboratory, Department of Public Health, Ministry of Health, Thimphu, Bhutanb; Department of Pediatrics, Jigme Dorji Wangchuk National Referral Hospital, Thimphu, Bhutanc; Research Promotion Institute, Oita University, Yufu, Japand

## Abstract

In addition to the eight genotypes of classic human astroviruses, seven new genotypes have been reported from two novel clades, MLB and VA. However, the epidemiology of these highly diverse astroviruses remains largely unknown. We report here the complete genome sequences of two MLB1 strains from Bhutanese children with diarrhea.

## GENOME ANNOUNCEMENT

Each year, 700,000 children aged <5 years die throughout the world due to diarrhea (1). Rotavirus is the most common cause of diarrhea, but the focus is now shifting to other viruses to understand their impact since the introduction of two successful rotavirus vaccines (2, 3). Astroviruses are responsible for 20% of diarrhea cases and have the potential for zoonosis because these highly diverse viruses infect a wide range of animals (4).

In 2009, astrovirus clade MLB1 was detected during an outbreak of diarrhea in Australian children (5). Subsequent studies revealed the presence of MLB1 among diarrheal patients in the United States, China, Hong Kong, Egypt, India, and Nigeria, although complete genome sequences of this virus are only available from Australia, United States, China, and Hong Kong. The epidemiology and spread of this virus remain largely unknown in developing countries, where diarrhea is the leading cause of mortality and morbidity (5–7). Bhutan is a small landlocked country in southeast Asia, where diarrhea is a public health concern and the causes of viral diarrhea have not been investigated. In this study, we report the complete genome sequences of two MLB1 astroviruses, which were detected by PCR (5) in Bhutanese children with diarrhea during viral diarrhea surveillance in children aged <5 years. Strain BtnMLB1-40 was detected in a 16-month-old boy who was an outpatient at the Jigme Dorji Wangchuk National Referral Hospital, Thimphu. Strain BtnMLB1-86 was detected in a 1-month-old boy who was admitted to Gelephu Regional Referral Hospital, Sarpang.

The complete genome sequence of MLB1 was determined using primers which were designed using sequences reported previously (7, 8) from China (GenBank accession no. JQ086552). The terminal parts were amplified using a rapid amplification of cDNA ends (RACE) kit, and the PCR products were purified and sequenced (9).

Astroviruses are nonenveloped viruses with a positive-sense RNA genome that belong to the family *Astroviridae*. The genome has three open reading frames (ORFs), ORF1a, ORF1b, and ORF2, which encode protease, RNA-dependent RNA polymerase, and capsid protein, respectively.

The complete genome sequences of Bhutanese MLB1s contain 6,171 nucleotides (nt), excluding the poly(A) tail. ORF1a contains 2,364 nt from nt 15 to 2378, ORF1b contains 1,536 nt from nt 2315 to 3859 nt, and ORF2 contains 2,268 nt from nt 3843 to 6110. Both strains have a 30-nt 5′-untranslated region and heptameric slippery sequence, which are conserved in astroviruses (7). The entire genome sequences of the Bhutanese strains share 99% identity, as well as 98%, 96%, 95%, and 92% identity with MLB1 strains from Hong Kong, China, Australia, and the United States, respectively. For the other MLB1 strains, the shared nucleotide (amino acid) identities with ORF1a, ORF1b, and ORF2 of the Bhutanese strains are 92 to 98% (98 to 99%), 94 to 99% (98 to 100%), and 91 to 98% (98 to 100%), respectively. The Bhutanese strains share their highest nucleotide and amino acid identities with the MLB1 strains from Hong Kong (8) and their lowest identities with the strains from the United States (5).

### Nucleotide sequence accession numbers.

The genome sequences of BtnMLB1-40 and BtnMLB1-86 appear in the DDBJ/EMBL/GenBank databases with accession no. AB823731 and AB823732, respectively.
